# Effect of *Akkermansia muciniphila* on pancreatic islet β-cell function in rats with prediabetes mellitus induced by a high-fat diet

**DOI:** 10.1186/s40643-024-00766-4

**Published:** 2024-05-19

**Authors:** Shuai Yan, Lin Chen, Na Li, Xiaohui Wei, Jingjing Wang, Weiping Dong, Yufan Wang, Jianxia Shi, Xiaoying Ding, Yongde Peng

**Affiliations:** 1 Department of Endocrinology and Metabolism, Shanghai General Hospital, Shanghai Jiao Tong University School of Medicine, Shanghai, 200080 China; 2 Shanghai Key Laboratory for Pancreatic Diseases, Institute of Translational Medicine, Shanghai General Hospital, Shanghai Jiao Tong University School of Medicine, Shanghai, 201620 China

**Keywords:** *Akkermansia muciniphila*, Prediabetes mellitus, β-cell function, Gut microbiome, Metaflammation, Toll-like receptors

## Abstract

Prediabetes is an important stage in the development of diabetes. It is necessary to find a safe, effective and sustainable way to delay and reverse the progression of prediabetes. *Akkermansia muciniphila* (*A. muciniphila*) is one of the key bacteria associated with glucose metabolism. Recent studies mainly focus on the effect of *A. muciniphila* on obesity and insulin resistance, but there is no research on the effect of *A. muciniphila* on pancreatic β-cell function and its mechanism in prediabetes. In this study, we investigated the effects of *A. muciniphila* on β-cell function, apoptosis and differentiation, as well as its effects on the gut microbiome, intestinal barrier, metaflammation and the expression of Toll-like receptors (TLRs) in a high-fat diet (HFD)-induced prediabetic rat model. The effect of *A. muciniphila* was compared with dietary intervention. The results showed both *A. muciniphila* treatment and dietary intervention can reduce metaflammation by repairing the intestinal barrier in rats with prediabetes induced by an HFD and improve β-cell secretory function, apoptosis and differentiation through signaling pathways mediated by TLR2 and TLR4. Additionally, *A. muciniphila* can further elevate β-cell secretion, attenuate apoptosis and improve differentiation and the TLR signaling pathway on the basis of diet.

## Graphical Abstract

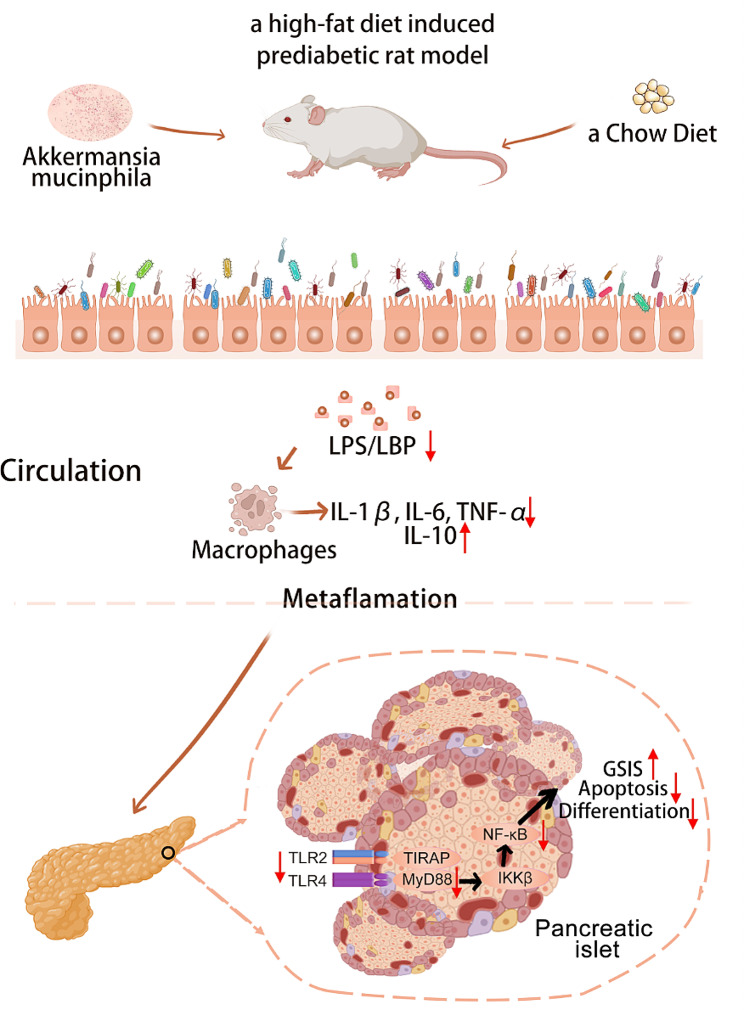

## Introduction

Prediabetes mellitus (pre-DM) includes impaired fasting glucose, impaired glucose tolerance or a combination of both. The global prevalence of pre-DM is 16.8% in 2021 and ever increasing (International Diabetes Federation 2021). In patients with pre-DM, the annual incidence of diabetes is from 5 to 10% and the cumulative incidence of diabetes is 95.9% in the past 30 years (Gong et al. 2019). People with pre-DM are at increased risk of macrovascular and microvascular diseases, tumors and dementia (Echouffo-Tcheugui and Selvin 2021). At present, numerous epidemiological studies and clinical trials for pre-DM have been conducted by the Daqing Research in China, the Diabetes Prevention Program in America, and the Diabetes Prevention Study in Finland. There is emerging evidence that lifestyle interventions and drug treatments can delay or reverse pre-DM (Gong et al. 2019; Tuomilehto et al. 2001; Knowler et al. 2002; Goldberg et al. 2009). However, due to the difficulty of lifestyle intervention and drug side effects, there is no definitive and universally effective treatment for pre-DM.

In the pre-DM stage, the patients already present with both insulin resistance (IR) and impaired insulin secretion from pancreatic islet β cells (Kahn 2001). Metaflammation is a chronic, low-grade inflammation that targets metabolically critical organs and tissues (Hummasti and Hotamisligil 2010). This systemic state is underlying pathophysiological basis of IR and β-cell dysfunction, and contributes to the occurrence and the progression of pre-DM and its complications (Gregor and Hotamisligil 2011; Rohm et al. 2022). The gut microbiome is referred to as the “second genome” of the human body, and it not only controls energy storage and metabolism but also regulates immune responses (Lloyd-Price et al. 2017). In a prospective cohort study, it was found that fecal microbiota of healthy individuals who go on to develop type 2 diabetes mellitus (T2DM) had already changed when they still were normoglycemic (Wang et al. 2021). Based on recent research, scientists have theorized that the gut microbiome, as a trigger for metaflammation, is directly involved in the pathogenesis of glucose metabolism disorders (Scheithauer et al. 2020; Tilg et al. 2020).

A growing number of studies investigating the intestinal flora of individuals with normal glucose tolerance, pre-DM, and T2DM have revealed distinct differences in the composition and abundance of intestinal flora between each population. Moreover, these studies have highlighted that specific bacterial species are associated with glucose and lipid metabolism, as well as inflammatory markers (Allin et al. 2018; Zhong et al. 2019; Karlsson et al. 2013; Wu et al. 2020). In the longitudinal multi-omics study of host–microbe dynamics in prediabetes, Zhou et al. found that the correlation of gut microbes was different in people with insulin sensitivity from IR, and there were different patterns of gut microbial interaction in the two groups (Zhou et al. 2019).

Recently, the use of probiotics to interfere with pre-DM has become a research hotspot. Traditional probiotics such as the bacteria of the genera *Lactobacillus* and *Bifidobacterium* are often used in these studies. Some studies show that supplementation of multiple probiotics or combined with synbiotics can improve glucose homeostasis and IR in patients with pre-DM (Zeighamy Alamdary et al. 2022). Nevertheless, the outcomes have yielded inconsistent results. Human observational studies on the relations between gut microbiota and glucose metabolism disorders have shown that *Akkermansia muciniphila* (*A. muciniphila*) is one of the key bacteria associated with glucose metabolism (Palmnäs-Bédard et al. 2022). *A. muciniphila* is a recently identified probiotic that is widely distributed throughout the intestinal mucus layer in mammals (Ottman et al. 2016). It can adhere to epithelial cells, increase the thickness of the intestinal mucosa, and strengthen the intestinal barrier (Reunanen et al. 2015). In recent studies, there is evidence that a significant decrease in the abundance of *A. muciniphila* in fecal samples of individuals with obesity, pre-DM, and diabetes. This reduction has been negatively correlated with blood glucose levels, blood lipids, and body weight (Allin et al. 2018; Zhong et al. 2019; Zhang et al. 2020). Following dietary intervention or treatment with drugs and surgery in individuals with obesity and T2DM, the abundance of *A. muciniphila* is restored, leading to improvements in metabolic activities (Dao et al. 2016; de la Cuesta-Zuluaga et al. 2017; Palleja et al. 2016). In animal studies, the treatment of *A.muciniphila* and its membrane protein have been reported to strengthen the intestinal barrier, alleviate endotoxemia, improve glucose and energy metabolism in obese and T2DM models (Everard et al. 2013; Zhang et al. 2018; Plovier et al. 2017), and reduce insulitis in NOD mice (Hänninen et al. 2018). Current studies mainly focus on the effect of *A. muciniphila* on obesity and IR, but there is a lack of research on the effect of *A. muciniphila* on β-cell function and its mechanism, especially in prediabetic subjects.

In this study, we investigated the effects of *A. muciniphila* on pancreatic β-cell function, apoptosis and differentiation, as well as its effects on the gut microbiome, intestinal barrier, metaflammation and the expression of Toll-like receptors (TLRs) in a high-fat diet (HFD)-induced prediabetic rat model. The effect of *A. muciniphila* was compared with dietary intervention to gain a deeper understanding of the role of *A. muciniphila* on β-cell function in pre-DM and its mechanism, with the ultimate goal of identifying effective approaches for preventing and treating pre-DM.

## Materials and methods

### Culture and preparation of *A. muciniphila*

*A. muciniphila* MucT (ATTC BAA-835) was anaerobically (85% nitrogen, 10% carbon dioxide, 5% hydrogen) cultured on a mucin-based medium and a synthetic medium in which mucin was replaced by a mixture of 16 g L^-1^ soy peptone, 4 g L^-1^ threonine, glucose and N-acetyl glucosamine (25 mmol L^-1^ each) (Plovier et al. 2017). Under strict anaerobic conditions, the culture was centrifuged at 10,000 × g for 20 min, washed, concentrated, and stored in anaerobic PBS containing 25% (v/v) glycerol at -80 ℃. Before intragastric administration, the stored bacterial cells were anaerobically thawed and diluted with anaerobic PBS to a final concentration of 2 × 10^10^ cfu mL^-1^ and 2.5% glycerol.

### Animals and treatment

#### Rat modeling

Fifty specific pathogen-free (SPF) male Sprague‒Dawley rats (6 weeks old, 200–250 g) were purchased from Shanghai SLAC Laboratory Animal Co., Ltd. (Shanghai, China). All animal experimental procedures were approved by the Institutional Animal Care and Use Committee of SLAC, Shanghai Laboratory Animal Center, Chinese Academy of Sciences (No. 20,200,120,019). All rats were raised under controlled conditions (12 h light / 12 h dark cycle, temperature 22 ± 1 ℃, humidity 55 ± 10%) at a density of two rats per cage. The rats were given rodent feed and sterilized water ad libitum in SPF housing in the laboratory. After one week of adaptive feeding of a chow diet, they were randomly divided into two groups. The control group (*n* = 10) was fed a chow diet (R39516908 carbohydrate 62%, 27% protein, and 11% fat, SLAC Inc. Shanghai, China), while the HFD group (*n* = 40) was fed a high-fat diet (HFD) (D12492 protein 20% carbohydrate, 20% protein, and 60% fat, Research Diets, Inc. New Brunswick, NJ, USA), and all of the rats were weighed once a week. After 16 weeks, an intraperitoneal glucose tolerance test (IPGTT) was performed with all rats. Fasting plasma glucose (FPG), 2-hour postload plasma glucose (2h-PG), and fasting serum insulin (FINS) were measured, and the homeostasis model assessment of insulin resistance (HOMA-IR) was calculated using the following equations:

$$HOMA-IR=\left(FPG(mmol/L)\times FINS(mIU/L)\right)/22.5$$.

The prediabetic rat model was established when FPG was between 6 and 7 mmol L^−1^ or 2h-PG was between 7.8 and 11.1 mmol L^− 1^, and body weight, FINS and HOMA-IR were significantly higher than those of the rats in the control group (Ren et al. 2022; Obrosova et al. 2007). 32 rats in the HFD group were considered to be the rats with pre-DM (*n* = 32).

#### Rat intervention

**Fig. 1:**
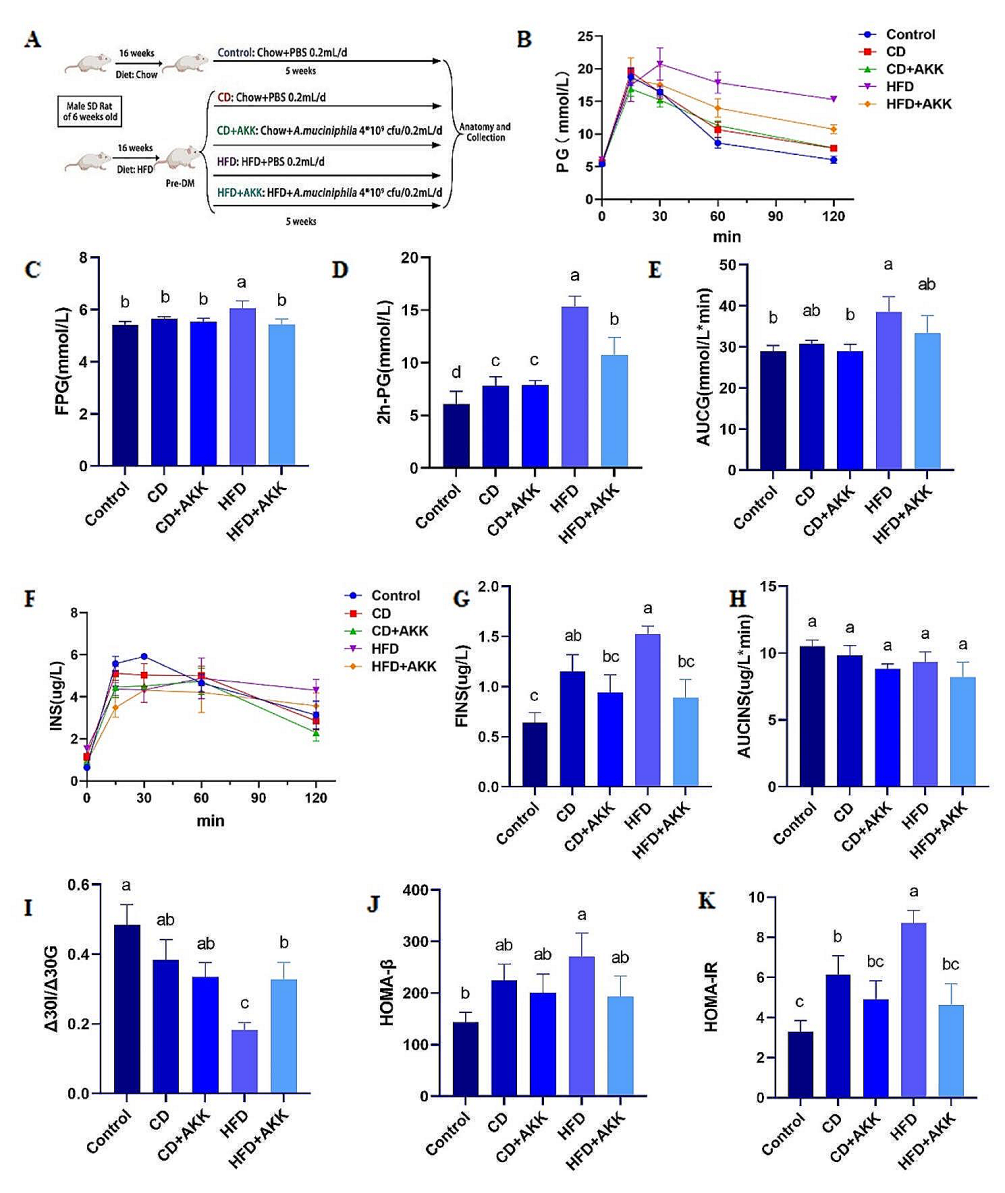
Effects of *A. muciniphila* treatment and dietary intervention on glucose metabolism and pancreatic islet function in the rats with pre-DM: **A** Schematic representation of the animal experiment process; **B** Changes in the blood glucose levels of rats in different groups during the IPGTT; **C-E** The levels of FPG, 2 h-PG and AUCG of rats in different groups during the IPGTT; **F** Insulin release curve of rats in different groups during IPGTT; **G-K** The levels of FINS, AUCINS, Δ I30/Δ G30, HOMA- β and HOMA-IR of rats in different groups during the IPGTT. Different letters in the same bar represent significant differences between the treatments when *P* < 0.05

The rats with pre-DM induced by an HFD were randomly divided into four groups (32 rats, *n* = 8 per group), two groups of which were switched back to a chow diet, and the other two groups were kept on an HFD. The CD + AKK and HFD + AKK groups were treated daily with an oral gavage of *A. muciniphila* at a dose 4 × 10^9^ cfu suspended in 0.2mL of sterile anaerobic PBS with different diets. The CD and HFD groups were treated daily with an oral gavage of an equivalent volume of sterile anaerobic PBS with different diets. The rats in the control group were kept on a chow diet and treated daily with an oral gavage of an equivalent volume of sterile anaerobic PBS (control group, *n* = 10). All rats were treated for 5 weeks and weighed once a week. The conversion from an HFD to a chow diet in rats with pre-DM was considered the dietary intervention. The model and treatment of the rats with pre-DM are shown in Fig. 1A.

#### Tissue sampling

After *A. muciniphila* treatment and dietary intervention for 5 weeks, the feces of all rats were collected under aseptic conditions and stored at -80 °C for further DNA extraction and analysis. IPGTT was performed in all rats. The levels of plasma glucose, serum insulin, lipopolysaccharide binding protein (LBP), IL-1β and IL-10 were measured, and the area under the curve of glucose and insulin (AUCG, AUCINS), insulin/glucose 30 min ratio (ΔI30/ΔG30), β cell function of homeostasis model assessment (HOMA-β) and HOMA-IR were calculated. Pancreatic islets were isolated and cultured. Glucose-stimulated insulin secretion (GSIS) was performed in each group. Islets and cell supernatants were collected and stored at -80 ℃ for further experiments. After the rats were killed by cervical dislocation, the pancreatic tissues and ileal tissues were fixed in 4% paraformaldehyde solution or stored in liquid nitrogen for further experiments.

### IPGTT

Before and at 15, 30, 60 and 120 min after intraperitoneal injection of 2 g kg^− 1^ glucose (50% dextrose), blood samples were obtained from the orbital sinus of all rats that had been fasted for 12 h. Blood glucose levels were monitored by a glucose monitor (Abbott, Chicago, IL, USA). The blood samples were placed at room temperature for 30 min, and then the upper serum was taken after centrifugation at 3000 × g for 20 min to determine the concentration of serum insulin. AUCG, AUCINS, ΔI30/ΔG30, HOMA-β and HOMA-IR were calculated using the following equations (Albareda et al. 2000):

$$\begin{aligned}AUCG & =0.25\times FPG(mmol/L)+0.5\times 15minPG\\ & \quad +0.75\times 30minPG+60minPG+0.5\times 120minPG\end{aligned}$$

$$\begin{aligned}AUCINS & =0.25\times FINS(mIU/L)+0.5\times 15minINS\\ & \quad +0.75\times 30minINS+60minINS+0.5\times 120minINS\end{aligned}$$

$$ \varDelta I30/\varDelta G30= \frac{30minINS-FINS\left(mIU/L\right)}{30minPG-FPG\left(mmol/L\right)}$$

$$ HOMA-\beta =20\times FINS(mIU/L)/\left(FPG(mmol/L)-3.5\right)$$

$$HOMA-IR=\left(FPG(mmol/L)\times FINS(mIU/L)\right)/22.5$$

### Isolation and culture of rat pancreatic islets

Rats were anesthetized by intramuscular injection of sodium pentobarbital (50 mg kg^-1^). The abdomen was opened to expose the pancreas and common bile duct. The common bile duct was cannulated with a 26-gauge needle, and its outlets to the duodenum and porta hepatis were closed with surgical clamps. 10 mL (1 mg mL^-1^) of collagenase P (Roche Applied Science, Indianapolis, IN, USA) were retrogradely injected through the common bile duct to distend the pancreas. The pancreas was removed to 6 mL of Hanks’ balanced salt solution, placed in a water bath at 38 °C for 12 min and shaken vigorously. Digestion was stopped by adding 30 mL Hanks’ balanced salt solution containing 20 mmol L^-1^ HEPES and 0.1% BSA (HHBS) at 4 ℃. The tissue suspension was filtered with a 20-mesh screen, washed with HHBS at 4 °C and centrifuged at 339 × g for 2 min. 10 mL of cell separation solution Histopaque 1190 (Sigma, Louis, MO, USA) was added to the tissue precipitate and mixed well. Then, 6 mL of cell separation solution Histopaque 1077 (Sigma, Louis, MO, USA) and HHBS were added to the tissue suspension in turn. The above suspension was centrifuged at 339 × g for 30 min. The islets on the interface of Histopaque 1077 and HHBS were absorbed, screened with a 100 μm cell filter and washed down from the cell filter with 2 mL of 1640 medium. After culturing at 37 °C for 6 h, the islets of the rats in each group were divided into three samples. Two of them were stored at -80 °C for real-time fluorescence quantitative polymerase chain reaction (qRT‒PCR) and Western blot analysis. One of them was used for the GSIS test.

### GSIS

After culturing in ordinary 1640 medium containing fetal bovine serum, Pen-Strep, and 11 mmol/L glucose for 6 h, the collected islets were washed twice in glucose-free 1640 medium. Twenty islets of similar size were handpicked in six copies and incubated with 1640 medium containing either 2.8 mmol/L glucose or 16.7 mmol/L glucose at 37 °C for 1 h. The supernatant and islets were collected to determine the concentration of insulin in the supernatant and the concentration of islet protein. The GSIS index was calculated as follows: GSIS index = high glucose-stimulated insulin concentration (insulin concentration / islet protein concentration in high-glucose medium) / low glucose-stimulated insulin concentration (insulin concentration / islet protein concentration in low-glucose medium).

### Enzyme-linked immunosorbent assay (ELISA)

The insulin levels in rat serum and cell supernatants were measured by a rat insulin ELISA kit (10-1250-01, Mercodia, Uppsala, Sweden). The concentrations of LBP, IL-1β and IL-10 in rat serum were measured with various species LBP ELISA kits (HK-503, Hycult Biotech, Uden, Netherlands), rat IL-1 beta/IL-1F2 Quantikine ELISA kits (RLB00-1, R & D Systems, Minneapolis, MN, USA) and rat IL-10 Quantikine ELISA kits (R1000, R & D Systems, Minneapolis, MN, USA).

### Histopathological analysis

The ileal tissues of rats were fixed in 4% paraformaldehyde solution and embedded in paraffin. Sections were cut and stained with hematoxylin and eosin. Histopathological analysis was performed under a light microscope. The results regarding the damage to the ileal mucosal barrier in rats were reviewed by pathologists.

### Terminal-deoxynucleotidyl transferase-mediated dUTP nick-end labeling (TUNEL) and immunofluorescence

After fixation in 4% paraformaldehyde for 24 h, the pancreatic tissue was routinely embedded in paraffin and sectioned. Then, it was dewaxed with xylene and dehydrated in gradient ethanol. Tissue sections were placed in a repair box filled with citric acid antigen repair buffer (pH 6.0) for antigen repair in a microwave oven. The sections were washed with PBS (pH 7.4) and blocked with 3% BSA at room temperature for 30 min. The sections were incubated with anti-insulin antibody (GB11334, 1:200, Servicebio, Wuhan, China) at 4 °C overnight. After washing with PBS (pH 7.4), the sections were incubated with fluorescent secondary antibody (GB21303, 1:300, Servicebio, Wuhan, China) at room temperature for 50 min. After washing with PBS (pH 7.4), the sections were incubated with an in situ cell death detection kit, POD (11,684,817,910, Roche Applied Science, Indianapolis, IN, USA) at 37 ℃ for 1 h, and the nuclei were re-stained with 4,6-diamino-2-phenyl indole (DAPI). After washing with PBS (pH 7.4), the sections were sealed with anti-fluorescence quenching solution. The apoptosis of islet β cells was observed and photographed under an upright fluorescence microscope (Nikon Eclipse C1, Tokyo, Japan). The number of TUNEL^+^ β cells and total β cells were randomly counted in three fields, and the percentage of TUNEL^+^ β cells was calculated using the following equation: the percentage of TUNEL^+^ β cells (%) = the number of TUNEL^+^ β cells/the number of total β cells × 100%.

### Gut microbiome sequencing

To determine the structure and function profile of the gut microbial community, we used Illumina high-throughput sequencing to sequence the 16S rRNA gene V3-V4 region of the gut microbiota in rat feces. Total bacterial DNA was extracted from rat fecal samples using the OMEGA Soil DNA Kit (M5635-02, Omega Bio-Tek, Norcross, GA, USA). PCR amplification of the bacterial 16S rRNA genes V3–V4 region was performed using the forward primer 338F (5’-ACTCCTACGGGAGGCAGCA-3’) and the reverse primer 806R (5’-GGACTACHVGGGTWTCTAAT-3’). Sample-specific 7-bp barcodes were incorporated into the primers for multiplex sequencing. PCR amplicons were purified with Vazyme VAHTSTM DNA Clean Beads (Vazyme, Nanjing, China) and quantified using a Quant-iT PicoGreen dsDNA Assay Kit (Invitrogen, Carlsbad, CA, USA). After the individual quantification step, amplicons were pooled in equal amounts, and paired-end 2 × 250 bp sequencing was performed using the Illumina MiSeq platform with a MiSeq Reagent Kit v3 at Shanghai Personal Biotechnology Co., Ltd. (Shanghai, China).

Microbiome bioinformatics was performed with QIIME2 2019.4 (Bolyen et al. 2019) with slight modification according to the official tutorials (https://docs.qiime2.org/2019.4/tutorials/). Briefly, raw sequence data were demultiplexed and then denoised with the DADA2 plugin (Callahan et al. 2016) to obtain the amplicon sequence variant (ASV) frequency data table. Alpha-diversity metrics and beta diversity were estimated using the diversity plugin, and samples were rarefied to 25,389 sequences per sample. Taxonomy was assigned to ASVs using the classify-sklearn naïve Bayes taxonomy classifier in the feature-classifier plugin (Bokulich et al. 2018) against the Greengenes Database 13.8.

Sequence data analyses were mainly performed using QIIME2 and R packages (v3.2.0). ASV-level alpha diversity indices were calculated using the ASV table in QIIME2 and visualized as box plots. ASV-level ranked abundance curves were generated to compare the richness and evenness of ASVs among samples. Beta diversity analysis was performed to investigate the structural variation of microbial communities across samples using UniFrac distance metrics (Lozupone et al. 2007) and visualized via principal coordinate analysis (PCoA) and nonmetric multidimensional scaling (NMDS) hierarchical clustering (Ramette 2007). The significance of differentiation of microbiota structure among groups was assessed by permutational multivariate analysis of variance (PERMANOVA) using QIIME2. The taxonomy compositions and abundances were visualized using MEGAN (Huson et al. 2011) and GraPhlAn (Asnicar et al. 2015). A Venn diagram was generated to visualize the shared and unique ASVs among samples or groups using the R package “VennDiagram” based on the occurrence of ASVs across samples/groups regardless of their relative abundance (Zaura et al. 2009).

### RNA preparation and qRT‒PCR analysis

Total RNA was extracted from islets. Subsequently, 1 µg of total RNA was reverse-transcribed into cDNA using a PrimeScript™ RT Reagent Kit with gDNA Eraser (RR047A, Takara, Kyoto, Japan). qRT‒PCR was then performed in duplicate using a SYBR Premix Ex Taq™ Kit (RR420A, Takara, Kyoto, Japan) and an Applied Biosystems QuantStudio 6 Flex machine (Thermo Fisher Scientific, Waltham, MA, USA). The primer sequences are listed in Table 1. The obtained results were normalized to those of β-actin, which was used as a reference gene. All primers were purchased from Sangon Biotech (Shanghai, China). The data were analyzed using the 2 (− delta delta Ct) method.

**Table 1:** Primer sequences for qRT‒PCR

Gene Name	Species	Primer Sequence (5’-3’)
TLR2	Rat	F: TGTTCCGGGCAAATGGATCA
		R: GCCTGAAGTGGGAGAAGTCC
TLR4	Rat	F: TATCGGTGGTCAGTGTGCTT
		R: CTCGTTTCTCACCCAGTCCT
MyD88	Rat	F: CGACGCCTTCATCTGCTACTGC
		R: CCACCACCATGCGACGACAC
NF-kB	Rat	F: TCCCCTGTACGATAGTCGGCTC R: GAGCGTTGCTTTGGATCAAGG
Caspase3	Rat	F: GAGACAGACAGTGGAACTGACGATG R: GGCGCAAAGTGACTGGATGA
Bcl-2	Rat	F: TACGAGTGGGATACTGGAGATGAAGAC
		R: TCGGTTGCTCTCAGGCTGGAAG
Bax	Rat	F: TTCATCGAGCCCAGCA
		R: CTCGCTCAGCTTCTTGGTC
MAFA	Rat	F: CACCATCACCATCATCACCACCAC
		R: TGACCTCCTCCTTGCTGAAGCC
MAFB	Rat	F: CAACGGTAGTGTGGAGGACC
		R: ACGCGTTTATACCTGCACGA
β-actin	Rat	F: ACCCACACTGTGCCCATCTATG
		R: AATGTCACGCACGATTTCCCT

### Western blot analysis

Proteins from islets were extracted in RIPA lysis buffer supplemented with a cocktail of 2% protease inhibitors and phosphatase inhibitors. After centrifugation at 4 ℃ and 12,000 × g for 15 min, the supernatants of the lysate were collected, and the protein concentrations were determined by BCA. Equal amounts of proteins were separated by 10% SDS‒PAGE and transferred to polyvinylidene difluoride membranes. After blocking with protein-free rapid blocking buffer (PS108P, Epizyme Biotech, Shanghai, China) at room temperature for 1 h, the membranes were incubated with different primary antibodies at 4 °C overnight, such as a MAFA rabbit monoclonal antibody (mAb) (1:1000, 79,737, Cell Signaling Technology, Danvers, MA, USA), a MAFB rabbit mAb (1:1000, 30,919, Cell Signaling Technology, Danvers, MA, USA), an Occludin mAb (1:1000, 33-1500, Invitrogen, Carlsbad, CA, USA), an anti-TLR2 antibody (1:500, ab209217, Abcam, Cambridge, UK), an anti-TLR4 antibody (1:500, ab13867, Abcam, Cambridge, UK), a GAPDH rabbit mAb (1:5000, 5174, Cell Signaling Technology, Danvers, MA, USA), and a beta actin polyclonal antibody (1:5000, 20536-1-AP, Proteintech, Chicago, IL, USA). After washing with TBST three times (10 min each), the blots were incubated with secondary antibodies at room temperature for 1 h, including peroxidase-conjugated AffiniPure goat anti-rabbit IgG (H + L) (1:5000, 111-035-003, Jackson ImmunoResearch, West Grove, PA, USA) and peroxidase-conjugated AffiniPure goat anti-mouse IgG (H + L) (1:2000, 115-035-003, Jackson ImmunoResearch, West Grove, PA, USA). Finally, the target proteins were visualized using Immobilon ECL Ultra Western HRP Substrate (WBULS0500, Millipore, Boston, MA, USA). GAPDH and beta actin were used as internal controls. Protein bands were quantified via densitometric analyses using ImageJ software.

### Statistical analysis

SPSS 25.0 (IBM, Armonk, NY, USA) was used for statistical analysis, and the data are expressed as the means ± standard deviations (SDs). The values between the two groups were compared by unpaired t test, and the values among multiple groups were compared by one-way analysis of variance (ANOVA) and Post-hoc LSD tests. *P* < 0.05 was considered to indicate statistical significance.

## Results

### Establishment of a prediabetic rat model

After 16 weeks of HFD, in 32 of the 40 rats in the HFD group, FPG was less than 7 mmol L^-1^ and 2h-PG was between 7.8 and 11.1 mmol L^-1^, and their body weight, FINS and HOMA-IR were significantly higher than those of the rats in the control group (*P* < 0.01) (Table 2). Those rats were considered to be the rats with pre-DM (*n* = 32), and the modeling rate of pre-DM was 80%.

The rats with pre-DM induced by an HFD were randomly divided into four groups (*n* = 8 per group). Before intervention, there were no significant differences in body weight, glucose metabolism index or islet function among the rats with pre-DM in each group (*P* > 0.05), but there were significant differences in those indices except FPG between the rats with pre-DM and the rats in the control group (*P* < 0.05 or *P* < 0.01) (Table 3).

**Table 2:** Comparison of glucose metabolism indices between the two groups of rats

	control (*n* = 10)	pre-DM (*n* = 32)	t value	*P* value
Weight (g)	647.8 ± 38.08	714.06 ± 72.22**	-2.732	0.009
FPG (mmol/L)	4.48 ± 0.70	4.77 ± 0.62	-1.236	0.224
2h-PG (mmol/L)	4.99 ± 0.77	9.30 ± 1.09**	-11.610	0.000
FINS (ug/L)	0.27 ± 0.09	1.59 ± 0.92**	-8.011	0.000
HOMA-IR	1.13 ± 0.39	7.23 ± 4.37**	-7.805	0.000

The data are presented as the means ± SDs for continuous data, **P < 0.01

**Table 3:** Comparison of baseline glucose metabolism indices of the rats between each prediabetic group and the control group

	control (*n* = 10)	CD (*n* = 8)	CD + AKK (*n* = 8)	HFD (*n* = 8)	HFD + AKK (*n* = 8)
Weight (g)	652.6 ± 33.78^a^	733.63 ± 87.08^b^	740.13 ± 93.39^b^	737.25 ± 101.37^b^	731.13 ± 74.69^b^
FPG (mmol/L)	4.48 ± 0.70^a^	4.95 ± 0.39^a^	4.96 ± 0.86^a^	4.55 ± 0.67^a^	4.61 ± 0.49^a^
2h-PG (mmol/L)	4.99 ± 0.77^A^	9.36 ± 1.12^B^	9.48 ± 1.37^B^	9.26 ± 0.93^B^	9.09 ± 1.06^B^
FINS (ug/L)	0.27 ± 0.09^A^	1.95 ± 0.89^B^	1.68 ± 1.27^B^	1.45 ± 0.87^B^	1.35 ± 0.50^B^
HOMA-IR	1.13 ± 0.39^A^	9.27 ± 4.36^B^	7.63 ± 5.61^B^	6.46 ± 4.16^B^	5.76 ± 2.03^B^

The data are presented as the means ± SDs for continuous data. Different letters in the same row represent significant differences between the groups when *P* < 0.05 or *P* < 0.01

### *A. muciniphila* delayed the progression of pre-DM

Following continuous intake of an HFD, FPG and 2h-PG levels in the rats with pre-DM were increased. Specifically, FPG levels significantly surpassed those in the control group (*p* < 0.05), and the 2h-PG levels exceeded 11.1 mmol/L, which was significantly higher than that in the other prediabetic groups (*p* < 0.01). However, following treatment with *A. muciniphila* or with dietary intervention, no significant differences in FPG levels were observed between the prediabetic groups and the control group (*p* > 0.05), and the 2h-PG levels observed in the prediabetic groups were within 7.8–11.1 mmol/L (Fig. 1B-D). Both treatment methods notably reduced the 120 min AUCG levels in the rats with pre-DM (*p* < 0.05) (Fig. 1E). These results suggest that *A. muciniphila* supplementation and dietary intervention are both effective methods for delaying the progression of pre-DM. Furthermore, *A. muciniphila* can also improve glucose metabolism in the rats with pre-DM under the condition of continuous HFD.

### *A. muciniphila* improved first-phase insulin secretion and IR in the rats with pre-DM

While none of the prediabetic groups displayed a significant difference in 120 min AUCINS values (*p* > 0.05), the ΔI30/ΔG30 levels in the prediabetic groups treated with *A. muciniphila* and a chow diet were significantly higher than those in the HFD group (*p* < 0.05) (Fig. 1F, H, I). Both *A. muciniphila* treatment and dietary intervention significantly reduced the FINS and HOMA-IR levels in the rats with pre-DM (*p* < 0.05 or *p* < 0.01). With respect to the HFD group, the CD + AKK group and the HFD + AKK group exhibited more significant reductions in FINS and HOMA-IR levels than the CD group *(p* < 0.01) (Fig. 1G, J, K). These findings suggest that both *A. muciniphila* treatment and dietary intervention can increase first-phase insulin secretion, reduce hyperinsulinemia, and alleviate IR in the rats with pre-DM. Moreover, *A. muciniphila* supplementation can further improve hyperinsulinemia and IR in the rats with pre-DM fed with either a chow diet or an HFD.

### *A. muciniphila* can promote insulin secretion and inhibit apoptosis and dedifferentiation in the β cells in rats with pre-DM

**Fig. 2:**
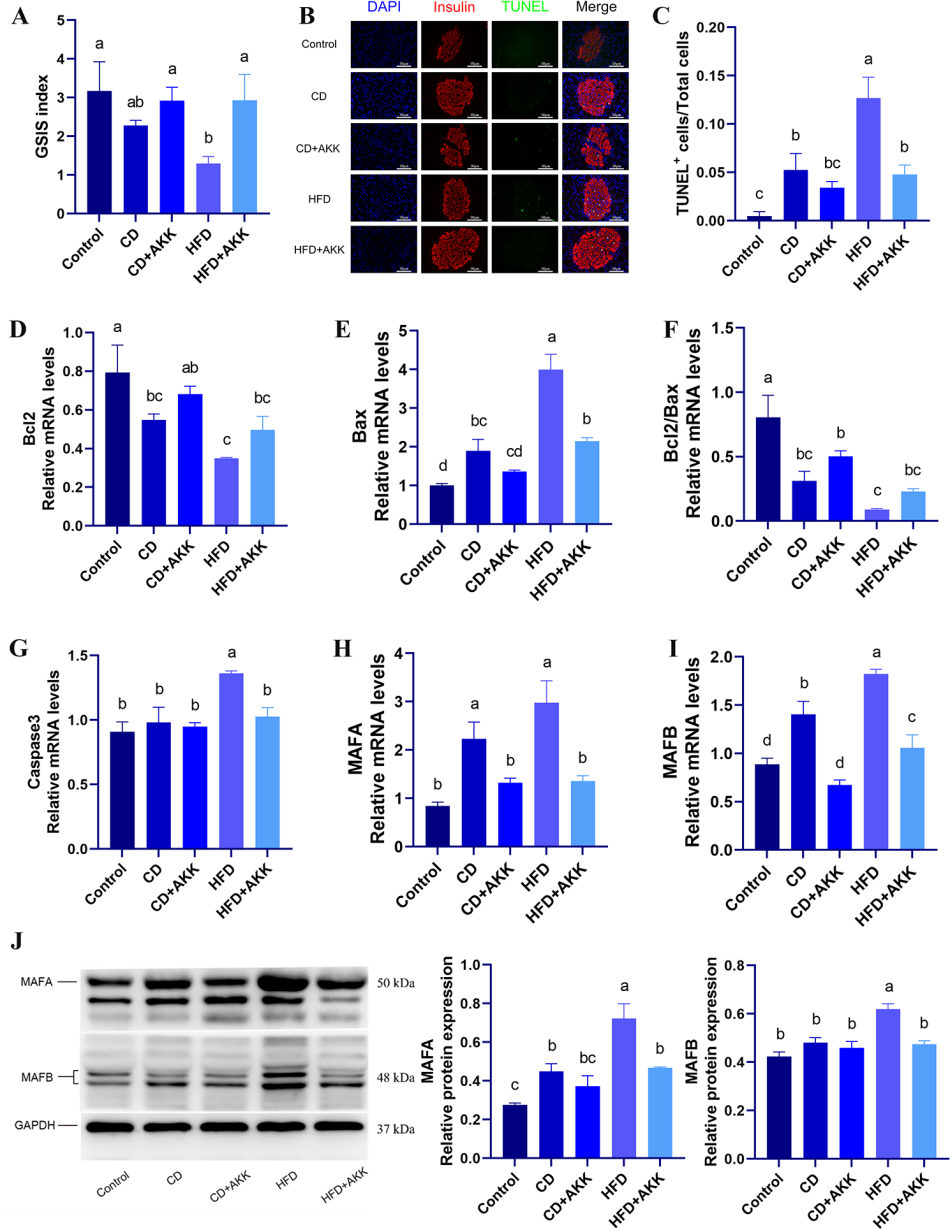
Effects of *A. muciniphila* treatment and dietary intervention on GSIS, apoptosis and differentiation in the β cells of rats with pre-DM: **A** GSIS index in the β cells of rats in different groups; **B** TUNEL and insulin immunofluorescence staining (×400) of the pancreatic tissues of rats in different groups (blue fluorescence is nuclear dye [DAPI] staining, red fluorescence represents insulin-positive β cells, green fluorescence represents TUNEL-positive apoptotic cells, and Merge is TUNEL combined with insulin immunofluorescence staining); **C** Comparison of the percentage of TUNEL^+^ β cells of rats in different groups; **D-I** Comparison of the expression of Bcl-2, Bax, Bcl-2/Bax, Caspase3, MAFA and MAFB at the mRNA level in the islets of rats in different groups; **J** Comparison of the expression of MAFA and MAFB at the protein level in the islets of rats in different groups. Different letters in the same bar represent significant differences between the treatments when *P* < 0.05

To assess the effects of *A. muciniphila* on insulin secretion, apoptosis, and differentiation in the β cells in rats with pre-DM, pancreatic islets and tissues were isolated from the rats in each group after 5 weeks *A. muciniphila* supplementation and dietary intervention. GSIS assays and TUNEL staining were performed, and the gene and protein expression of specific transcription factors related to β-cell apoptosis and differentiation was quantified. Following *A. muciniphila* supplementation or dietary intervention, the GSIS index in the rats with pre-DM surpassed that in the HFD group, with the more substantial increases in GSIS indices observed in the CD + AKK and HFD + AKK groups (*p* < 0.05) (Fig. 2A). These findings suggest that while both *A. muciniphila* treatment and dietary intervention can improve GSIS in the β cells in rats with pre-DM, *A. muciniphila* treatment can further restore GSIS in the rats with pre-DM fed with either a chow diet or an HFD.

Both *A. muciniphila* treatment and dietary intervention significantly reduced the percentage of TUNEL^+^ β cells in the pancreatic tissues of rats with pre-DM, and the more notable reductions were observed in the CD + AKK groups (*p* < 0.01) (Fig. 2B, C). Bcl-2 is an antiapoptotic factor, while Bax plays a role in promoting apoptosis. The higher the ratio of Bcl-2/Bax is, the stronger the antiapoptotic ability is. After both *A. muciniphila* supplementation and dietary intervention, upregulated the mRNA expression of Bcl-2 in the islets of rats with pre-DM was observed and significantly elevated expression levels were observed in the CD + AKK group compared with the HFD group *(p* < 0.01) (Fig. 2D). The mRNA expression levels of Bax were significantly lower in the islets of rats in the CD group, CD + AKK group and HFD + AKK group than that in the HFD group, especially in the CD + AKK group *(p* < 0.01) (Fig. 2E). At the levels of mRNA expression in the islets, the ratio of Bcl-2/Bax was also observed at the same trend as Bcl-2. The ratio of Bcl-2/Bax at the mRNA expression levels in the islets of rats in the CD group, CD + AKK group and HFD + AKK group was higher than that in the HFD group, significantly in the CD + AKK group *(p* < 0.01) (Fig. 2F). Furthermore, *A. muciniphila* supplementation and dietary intervention both downregulated the mRNA expression of the proapoptotic factor Caspase3 in the islets of rats with pre-DM (*p* < 0.01) (Fig. 2G). These findings suggest that *A. muciniphila* treatment and dietary intervention can both inhibit β-cell apoptosis in the rats with pre-DM, while *A. muciniphila* can further exert antiapoptotic effects on β cells in the rats with pre-DM fed with either a chow diet or an HFD. Moreover, treatment combined with *A. muciniphila* and a chow diet had a more pronounced antiapoptotic effect on β cells.

The gene and protein expressions of MAFA, which is a marker of β-cell maturation, and MAFB, which is a marker of β-cell dedifferentiation, were significantly downregulated in the islets of rats with pre-DM following both *A. muciniphila* treatment and dietary intervention, particularly in those of the CD + AKK and HFD + AKK groups *(p* < 0.01) (Fig. 2H-J). These findings suggest that *A. muciniphila* treatment and dietary intervention can hinder the maturation of β cells and inhibit the dedifferentiation of β cells into α cells in rats with pre-DM. Additionally, there is the further inhibitory effect of *A. muciniphila* on β-cell dedifferentiation in the rats with pre-DM fed with either a chow diet or an HFD.

### Effects of dietary intervention and *A. muciniphila* treatment on the intestinal flora and intestinal mucosal barrier in rats with pre-DM

**Fig. 3:**
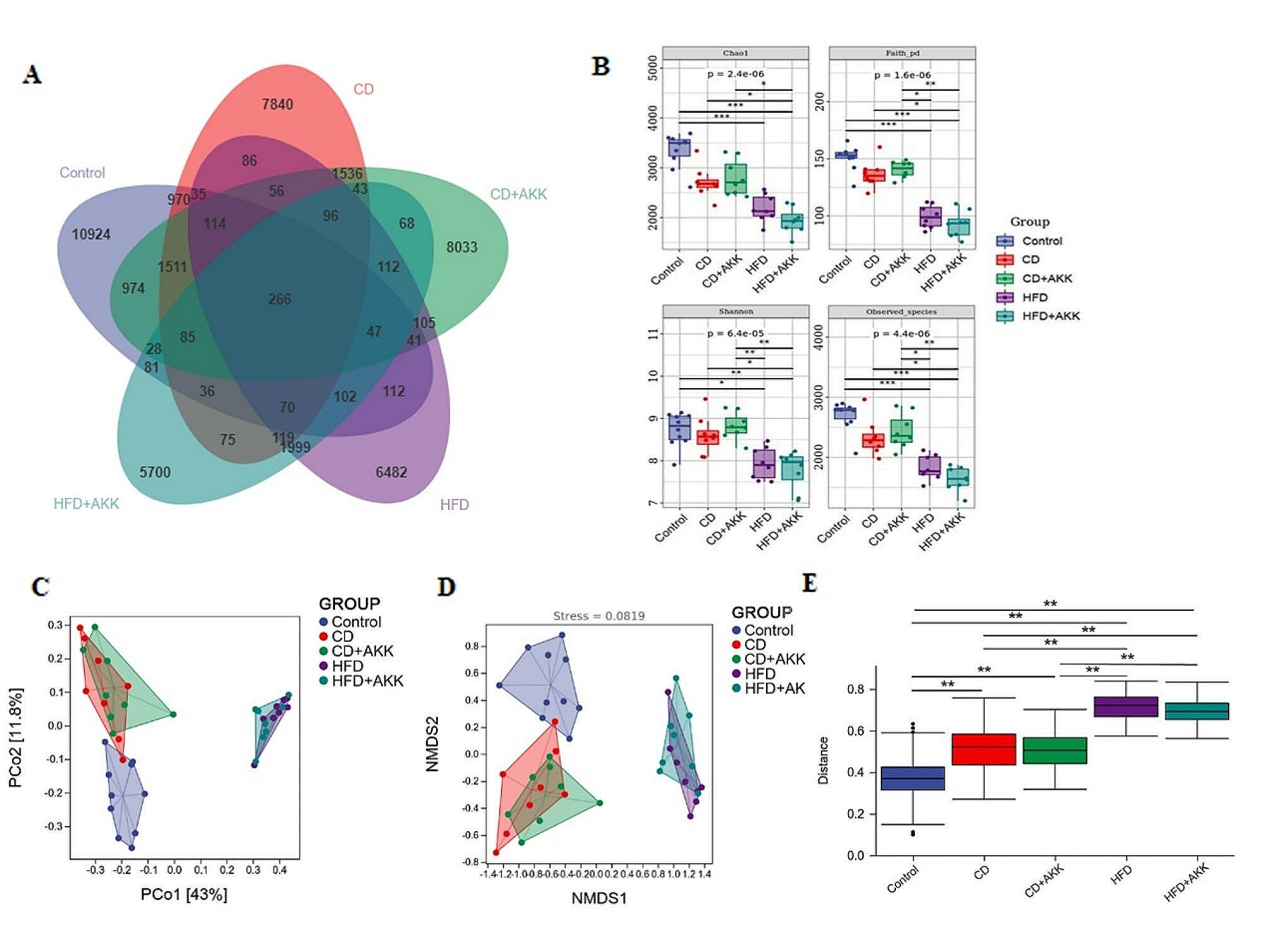
Comparison of the gut microbiome diversity of rats in different groups: **A** Venn diagrams at the ASV level of rats in different groups; **B** Alpha diversity index (Chao1, Faith’s PD, Shannon and observed species) values of the gut microbiome of rats in different groups; **C-D** Beta diversity analysis of the gut microbiome of rats in different groups determined using PCoA and NMDS based on weighted UniFrac distance; **E** Box diagram of the weighted UniFrac distance in different groups. * *P* < 0.05, ** *P* < 0.01, *** *P* < 0.001

After *A. muciniphila* treatment and dietary intervention for 5 weeks, DNA fragments extracted from the fecal samples of the rats in each group were sequenced with the Illumina platform. The Venn diagram plot of the intestinal flora illustrated that the number of specific ASVs in the HFD group was less than that in the control group. Dietary intervention increased the quantity of specific ASVs in the intestinal flora of rats with pre-DM, while *A. muciniphila* treatment had a minimal effect on the quantity of ASVs (Fig. 3A). Alpha diversity indices, such as Chao1 richness estimator, Faith’s PD, Shannon diversity index, and Observed species in the gut microbiome in the control group and each prediabetic group were analyzed (Fig. 3B). The beta diversity of the gut microbiome in each group was analyzed by PCoA and NMDS based on the weighted UniFrac distance and compared among the groups by PERMANOVA (Fig. 3C-E). These data suggest that dietary intervention can significantly improve the alpha diversity and beta diversity of the gut microbiome in rats with pre-DM (*p* < 0.05, *p* < 0.01 or *p* < 0.001), while *A. muciniphila* supplementation has no observable effects under the same dietary conditions.

**Fig. 4:**
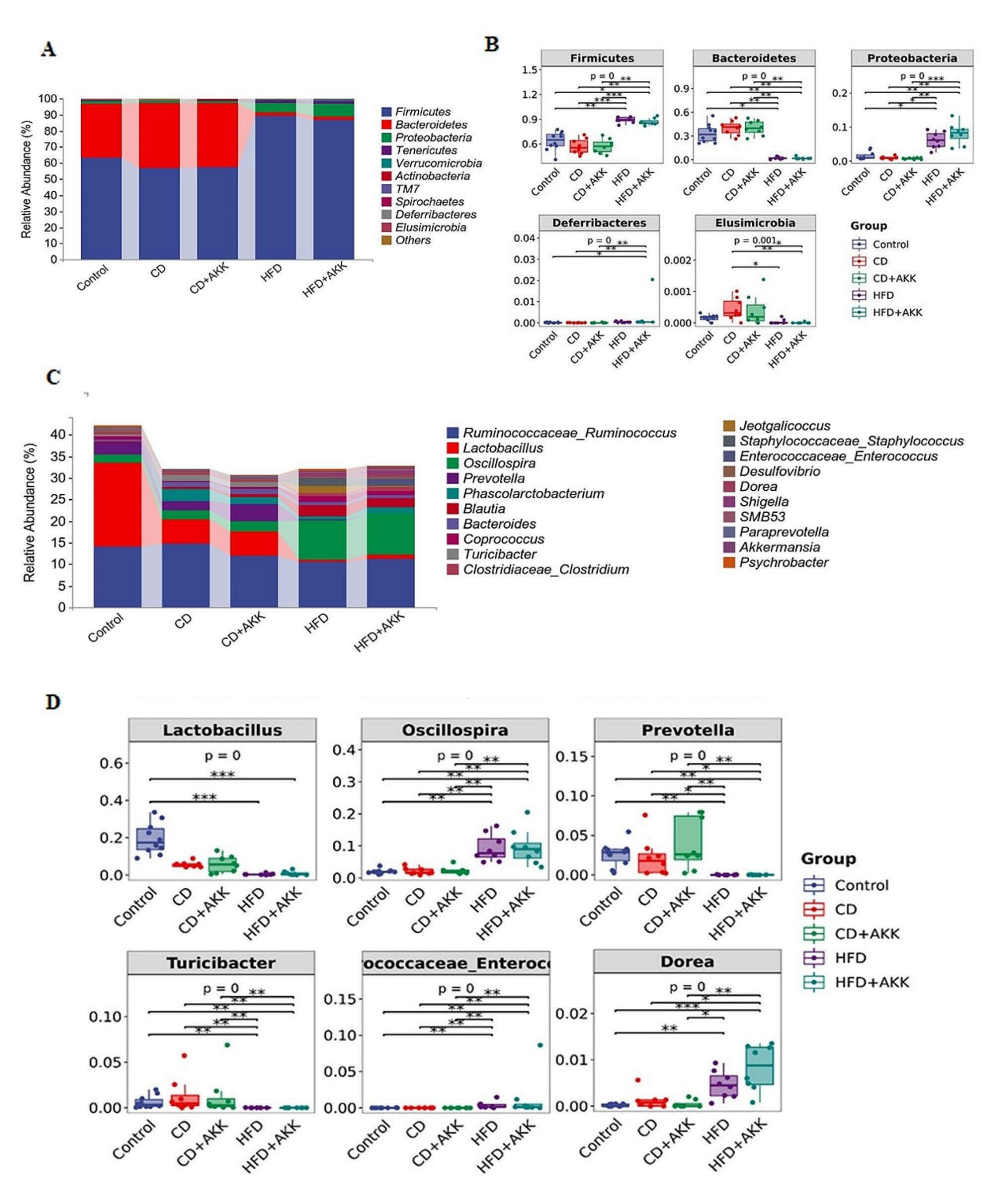
Bacterial community relative abundance of the rats in different groups: **A-B** At the phylum level, the composition and abundance of the top 10 flora in terms of relative abundance of the rats in different groups; **C-D** At the genus level, the composition and abundance of the top 20 flora in terms of relative abundance of the rats in different groups. * *P* < 0.05, ** *P* < 0.01, *** *P* < 0.001

At the phylum level, the dominant constituents of the gut microbiota in our rat model included *Firmicutes* (70.77%), *Bacteroides* (23.5%), and *Proteobacteria* (3.51%) (Fig. 4A). Dietary intervention significantly decreased the relative abundance of *Firmicutes*, *Proteobacteria*, and *Deferribacteres* in the intestinal flora of the HFD-induced prediabetic rats while increasing the relative abundance of *Bacteroidetes* and *Elusimicrobia* (*p* < 0.05, *p* < 0.01 or *p* < 0.001). *A. muciniphila* had little effect on the relative abundance of gut microbes in the rats with pre-DM (Fig. 4B). At the genus level, the predominant components of the gut microbiota in our rat model were *Ruminococcus* (12.5%), *Lactobacillus* (6.42%), *Oscillospira* (5.04%), *Prevotella* (1.75%), *Phascolarctobacterium* (1.36%), and *Blautia* (1.27%) (Fig. 4C). We compared the differences in relative abundance of the top 20 genera in the intestinal flora of each group. Dietary intervention resulted in a significant increase in the relative abundance of *Lactobacillus*, *Prevotella*, and *Turicibacter* in the HFD-induced prediabetic rats, while the relative abundance of *Oscillospira*, *Enterococcus*, and *Dorea* decreased significantly (*p* < 0.05 or *p* < 0.01). *A. muciniphila* had a minimal effect on the relative abundance of the intestinal flora among the groups fed the same diets (Fig. 4D).

**Fig. 5:**
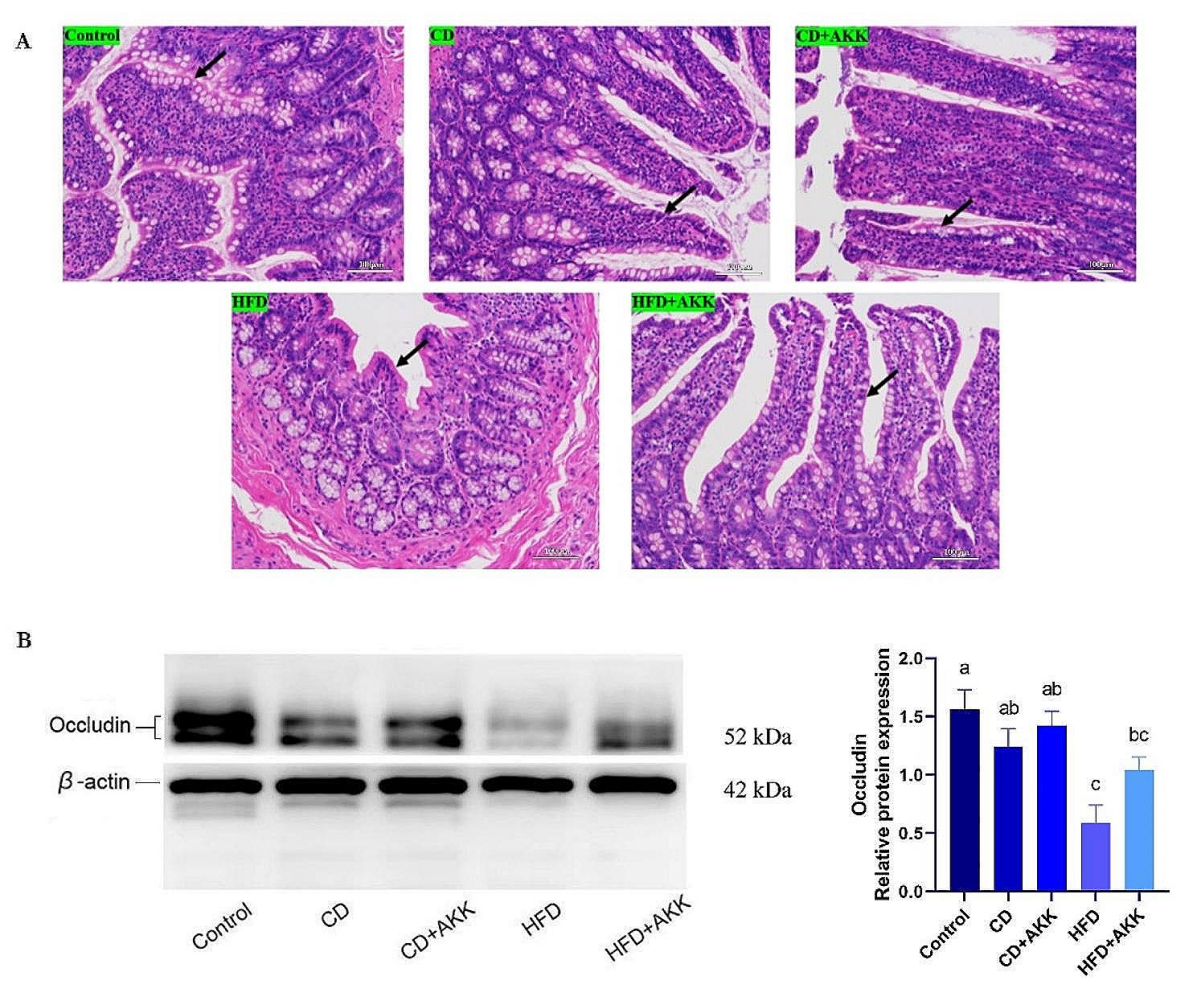
Effects of *A. muciniphila* treatment and dietary intervention on the intestinal barrier in rats with pre-DM: **A** HE staining in the ileum of rats in different groups (×200); **B** Expression of occludin at the protein level in the ileum of rats in different groups. Different letters in the same bar represent significant differences between the treatments when *P* < 0.05

Histopathological analysis of the ileum revealed that the villi in the HFD group were notably atrophied, which was evident by the shortened and rounded morphologies. Moreover, the quantity of goblet cells in the ileum decreased significantly. Although the quantity of goblet cells in the HFD + AKK group decreased only slightly, a similar degree of villous atrophy was observed in comparison with the HFD group. In the ileal mucosa in the CD and CD + AKK groups, significantly less villi damage was observed, and the number of goblet cells increased significantly compared with those in the HFD group (Fig. 5A). Dietary intervention in the HFD-induced prediabetic rats significantly upregulated the protein expression of occludin (*p* < 0.05), which is an enzyme involved in the formation of tight junctions in the ileal mucosa (Fig. 5B).

Therefore, dietary intervention significantly improved the composition and diversity of the gut microbiome and repaired the intestinal barrier in the rats with pre-DM, while *A. muciniphila* supplementation alone had little effect.

### *A. muciniphila* can reduce the levels of metaflammation in the circulation of rats with pre-DM

**Fig. 6:**
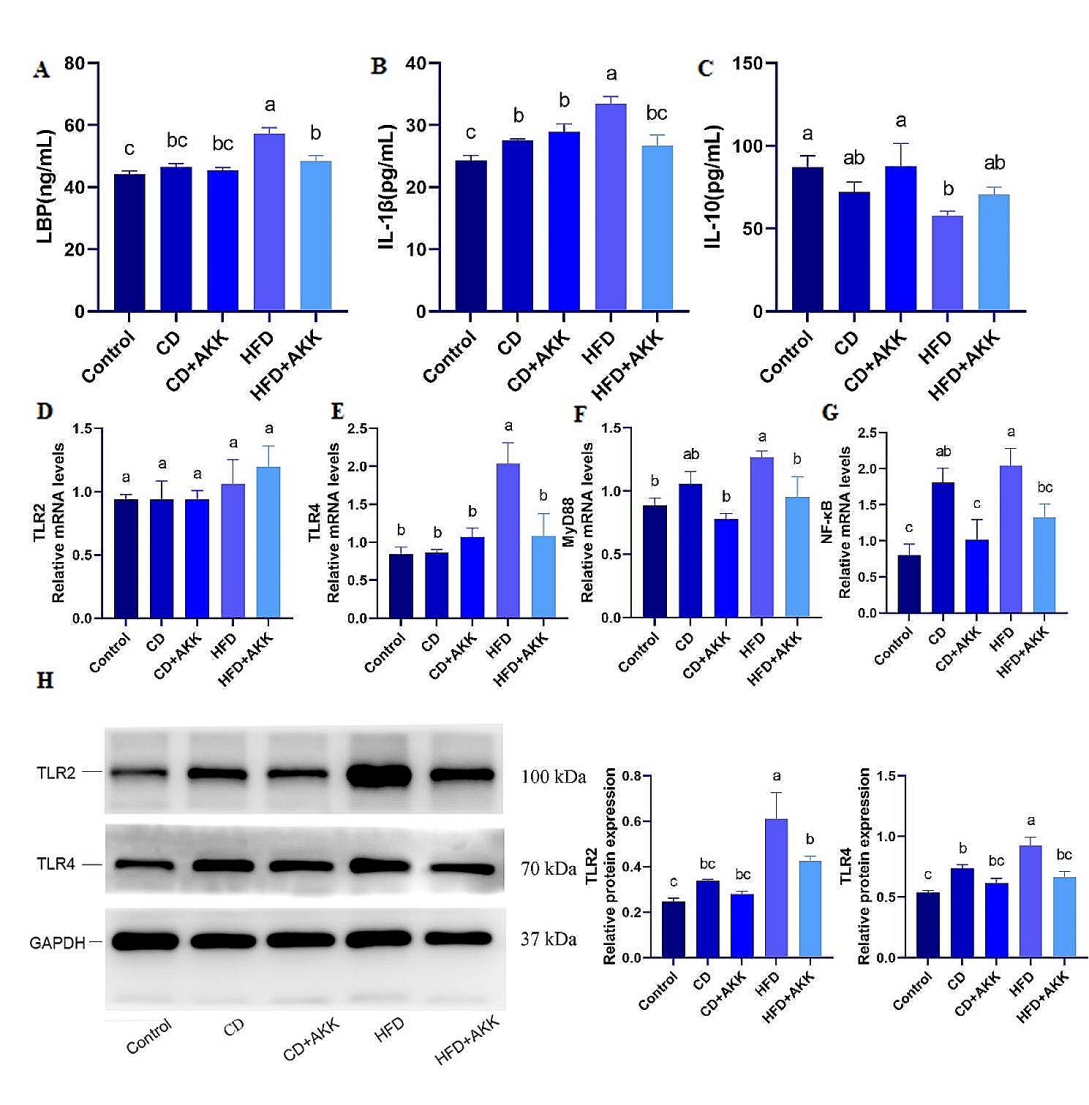
Effects of *A. muciniphila* treatment and dietary intervention on metaflammation and TLR signaling in islets in the rats with pre-DM: **A-C** Levels of serum LBP, IL-1β and IL-10 of the rats in different groups; **D-G** Comparison of the expression of TLR2, TLR4, MyD88 and NF-κB at the mRNA level in islets of rats in different groups; H: Comparison of the expression of TLR2 and TLR4 at the protein level in islets of rats in different groups. Different letters in the same bar represent significant differences between the treatments when *P* < 0.05

*A. muciniphila* treatment and dietary intervention both significantly reduced the serum levels of the proinflammatory cytokines LBP and IL-1β in rats with pre-DM (*p* < 0.01) (Fig. 6A, B). *A. muciniphila* treatment in conjunction with dietary intervention increased serum levels of the anti-inflammatory cytokine IL-10 in rats with pre-DM (*p* < 0.05) (Fig. 6C).

### *A. muciniphila* can inhibit the TLR2 and TLR4 signaling pathways in the pancreatic islets of rats with pre-DM

Following *A. muciniphila* treatment and dietary intervention for 5 weeks, there was no significant difference in TLR2 gene expression in the islets of rats between groups (*p* > 0.05) (Fig. 6D). Both *A. muciniphila* supplementation and dietary intervention significantly downregulated the protein expression of TLR2 in the islets of HFD-induced prediabetic rats, while the effect of dietary intervention was more pronounced (*p* < 0.05 or *p* < 0.01) (Fig. 6H). Each treatment significantly downregulated the gene and protein expression of TLR4 in the islets of rats with pre-DM (*p* < 0.05 or *p* < 0.01) (Fig. 6E, H). *A. muciniphila* inhibited the gene expression of the inflammatory pathway TLR4/MyD88/NF-κB in the islets of rats with pre-DM (*p* < 0.05). *A. muciniphila* supplementation combined with dietary intervention resulted in a more pronounced inhibitory effect on the TLR4 inflammatory pathway (*p* < 0.01) (Fig. 6E-H). These findings suggest that the gut microbiota may contribute to the regulation of β-cell apoptosis, differentiation, and function in rats with pre-DM through inflammatory pathways mediated by TLR2 and TLR4.

## Discussion

Pre-DM is an important stage in the development of diabetes. Recently, many studies have focused on the use of traditional probiotics to interfere with pre-DM, such as *Lactobacillus* and *Bifidobacterium*. Some studies have shown that supplementation of multiple probiotics or combined with synbiotics can improve glucose homeostasis and IR in the prediabetic patients, but the efficacy is not consistent (Zeighamy Alamdary et al. 2022). *A.muciniphila* is one of the next-generation probiotics. The decreased abundance of *A.muciniphila* in the intestine is closely related to the disorder of glucose metabolism (Palmnäs-Bédard et al. 2022). In this study, the effect of *A. muciniphila* was compared with dietary intervention in a prediabetic model induced by an HFD. It was found that *A. muciniphila* supplementation and dietary intervention both delayed the progression of pre-DM. Furthermore, *A. muciniphila* also improved glucose metabolism in the rats with pre-DM under the condition of continuous HFD. There were both impaired first-phase insulin secretion and insulin resistance in the rats with pre-DM induced by an HFD. Both *A. muciniphila* treatment and dietary intervention increased first-phase insulin secretion, and alleviated hyperinsulinemia and IR in the rats with pre-DM.

Growing evidence has shown the beneficial effect of *A. muciniphila* on obesity and IR (Everard et al. 2013; Zhang et al. 2018; Plovier et al. 2017), but there is no related research on the effect of the gut microbiome on β-cell function in pre-DM. Only some studies have found that *A. muciniphila* alleviates islet inflammation in NOD mice (Hänninen et al. 2018), increases the expression of insulin secretion genes and decreases the expression of apoptosis genes in INS-1 cells in vitro (Liu et al. 2020). In our work, the effect of *A. muciniphila* on the function of β cells in pre-DM were observed for the first time by isolating the pancreatic islets and tissues in each prediabetic group induced by an HFD and the control group after *A. muciniphila* treatment and dietary intervention. In a study of the dynamic pathophysiology of islet dysfunction and IR in diabetic mice induced by an HFD, Gao et al. found that there was transient compensatory proliferation in β cells in the first 8 weeks and decompensation of function and an increase in apoptosis and immune inflammation in β cells 12 weeks later (Gao et al. 2021). Therefore, we compared the secretory function, apoptosis and differentiation of β cells in each prediabetic group and the control group after *A. muciniphila* treatment and dietary intervention. The results demonstrated while both *A. muciniphila* supplementation and dietary intervention improved GSIS in the β cells in rats with pre-DM, *A. muciniphila* treatment further restored GSIS in the rats with pre-DM fed with either a chow diet or an HFD. It was also found that *A. muciniphila* supplementation and dietary intervention both inhibited β-cell apoptosis in the rats with pre-DM, and *A. muciniphila* further exerted antiapoptotic effects on β cells on the basis of diet. Moreover, *A. muciniphila* supplementation and dietary intervention had synergistic effect. Bcl-2 family regulates the mitochondrial apoptosis pathway through interaction among its members, which can activate Caspase3 mediated cascade in the downstream and cause apoptosis (Hanahan and Weinberg 2011). Our results showed that both *A. muciniphila* treatment and dietary intervention significantly downregulated the gene expression of Bax and Caspase3 in the islets of rats with pre-DM, and *A. muciniphila* supplementation and dietary intervention had synergistic effect on upregulation of the gene expression of Bcl-2 and the ratio of Bcl-2/Bax. The activity of these functional proteins will be determined at the protein level in future studies. In addition to proliferation and apoptosis, it has also been found that impairment of β-cell function is related to dedifferentiation, transdifferentiation and redifferentiation from β cells to other cells inside or outside the islets (Jeffery and Harries 2016; Brereton et al. 2016). Our findings showed that the expression of MAFA, which is a marker of β-cell maturation, was significantly upregulated in the islets of rats with pre-DM induced by an HFD. This might be related to the compensatory proliferation of β cells for increased insulin demand caused by IR and increased β-cell apoptosis in pre-DM. Both *A. muciniphila* supplementation and dietary intervention downregulated the expression of MAFA in the islets of rats with pre-DM, suggesting that differentiation and maturation of β cells decreased. This might be due to the improvement of IR and the inhibition of β-cell apoptosis after treatments. At the same time, both *A. muciniphila* treatment and dietary intervention also downregulated the expression of MAFB in the islets of rats with pre-DM, suggesting that β-cell dedifferentiation was inhibited. Moreover, there was the further inhibitory effect of *A. muciniphila* on β-cell dedifferentiation in the rats with pre-DM fed with either a chow diet or an HFD.

To further explore the possible mechanism of the effect of *A. muciniphila* on the function of β cells in pre-DM, we observed the differences of intestinal flora and the changes of intestinal barrier between each prediabetic group and the control group after *A. muciniphila* treatment and dietary intervention. The analysis of intestinal flora demonstrated that dietary intervention attenuated the decrease in diversity and reshaped the structure of the gut microbiome in rats with pre-DM induced by an HFD, while the effect of *A. muciniphila* alone was not obvious. In the assessment of intestinal barrier in the rats with pre-DM, the villi of the ileal mucosa were damaged and the number of goblet cells and the expression of Occludin in the intestinal mucosa were decreased significantly. Both *A. muciniphila* supplementation and dietary intervention increased the number of goblet cells and upregulated the expression of Occludin, but the effect of dietary intervention was more significant. Dietary intervention also improved the damaged intestinal villi, while *A. muciniphila* supplementation alone had little effect. This was the same as the effect of dietary intervention and *A. muciniphila* treatment on the intestinal flora, indicating that the effect of dietary intervention on the intestine is better than that of *A. muciniphila* alone. This may be related to the complexity and variability of the gut microbiome and the selection of experimental animal models.

When the intestinal mucosal barrier is damaged, the permeability of the intestine increases. The metabolites of bacteria and nutrients in the intestine are translocated into the circulation. Among them, lipopolysaccharide (LPS), which is a bacterial component, is an important inflammatory substance. An increased level of LPS in the circulation promotes the release of a variety of inflammatory factors by immunocytes and causes metaflammation in the body (Akira et al. 2006; Cani et al. 2007). A similar pattern of results was obtained in the rats with pre-DM induced by an HFD. Both *A. muciniphila* treatment and dietary intervention significantly reduced the levels of serum LBP and IL-1β in the rats with pre-DM, while the level of IL-10 was increased after dietary intervention combined with *A. muciniphila* supplementation. These findings suggest that both *A. muciniphila* supplementation and dietary intervention alleviate metaflammation in the rats with pre-DM. In view of the possible mechanism of the effects of *A. muciniphila* on islet function in pre-DM, we also detected the expression of LPS-recognizable TLRs in pancreatic islets. In TLRs, TLR2 and TLR4 are most closely related to metabolic diseases (Jialal et al. 2014; Dasu et al. 2010; Könner and Brüning 2011). Previous studies have found that LPS impairs function and affects survival in human β cells through TLR4 in vitro (He et al. 2019). Knockout of TLR4 in obese mice restores β-cell function (Yan et al. 2020). The impairment of β-cell function and insulitis caused by an HFD are also avoided in TLR2-knockout mice (Ehses et al. 2010). Combined knockout of TLR2 and TLR4 increases the compensatory proliferation of β cells in mice fed an HFD (Ji et al. 2019). Therefore, in our study, the expression levels of TLR2 and TLR4 and their related signaling pathways in islets were compared among the prediabetic groups and the control group. The expression of TLR2 and TLR4 was significantly upregulated in the islets of rats with pre-DM and was significantly downregulated after both *A. muciniphila* treatment and dietary intervention. Dietary intervention had a greater effect on the expression of TLR2, while *A. muciniphila* further inhibited the expression of TLR4 on the basis of diet. Additionally, *A. muciniphila* inhibited the gene expression of the inflammatory pathway TLR4/MyD88/NF-κB in the islets of rats with pre-DM, and *A. muciniphila* supplementation and dietary intervention had synergistic effect. These findings suggest that the intestinal flora may play a role in regulating the apoptosis, differentiation and function of β cells in rats with pre-DM through inflammatory signaling pathways mediated by TLR2 and TLR4. The activity detection of molecular proteins in the signaling pathway needs to be verified for further research.

In this study, it was found that compared with dietary intervention, *A. muciniphila* alone had little effect in the intestine, but it further improved the secretion, apoptosis, differentiation of β cells and the TLR signaling pathway in the pancreatic islets of rats with pre-DM on the basis of diet. The possible mechanism includes the following aspects to be confirmed by future research. (1) *A. muciniphila* can exert the beneficial effects on the host by its cell envelope components (Garcia-Vello et al. 2022; Bae et al. 2022), extracellular and secreted proteins (Plovier et al. 2017; Yoon et al. 2021; Cani and Knauf 2021) and metabolites, especially short-chain fatty acids. There are short-chain fatty acid receptors (FFA2 and FFA3) on pancreatic β cells, which can combine with short-chain fatty acids to regulate GSIS, β-cell mass and β cell responses to insulin resistance (Tang et al. 2015; McNelis et al. 2015). *A. muciniphila* mainly produces acetate and propionic acid from mucus (Derrien et al. 2004). It has been suggested that propionate can improve β-cell function in humans and stimulate insulin secretion from human islets in vitro (Pingitore et al. 2017). Thus, *A.muciniphila* might regulate the quantity and quality of β cells in pre-DM by binding its metabolites short-chain fatty acids to receptors on pancreatic islets, which will be further investigated. (2) *A. muciniphila* can also upregulate short-chain fatty acids in the gut through the metabolic interactions between *A. muciniphila* and butyrogenic bacterial taxa, perhaps especially butyrate (Belzer et al. 2017). Butyrate has been extensively shown to afford protection in pancreatic islet β cells (Pedersen et al. 2024). Whether the effects of *A. muciniphila* regulated butyrate are mediated via the upregulation of β-cell sirtuin-3 (Zhang et al. 2016), thereby optimizing pancreatic β-cell mitochondrial function and decreasing oxidant production will be important to clarify (Anderson and Maes 2020). These authors also highlight the butyrate upregulation of acetyl-CoA, which not only enhances mitochondrial ATP production but also acts as a necessary cosubstrate for the initiation of the mitochondrial melatonergic pathway (Anderson and Maes 2020). Exogenous melatonin is an established regulator of more optimized pancreatic β cells in the course of induced diabetes (Alaa et al. 2023), suggesting that its regulation in pancreatic β cells, as well as its exogenous efflux from other cells in the human pancreatic islet β-cell microenvironment (Anderson 2023), will be important to clarify.

## Conclusion

In conclusion, both *A. muciniphila* treatment and dietary intervention can reduce metaflammation by repairing the intestinal barrier in rats with pre-DM induced by an HFD and improve β-cell secretory function, apoptosis and differentiation through signaling pathways mediated by TLR2 and TLR4. Compared with dietary intervention, *A. muciniphila* supplementation alone has little effect on reshaping the intestinal flora structure and repairing the intestinal mucosal barrier in rats with pre-DM but has the same effect on improving islet β-cell function, alleviating metaflammation and inhibiting the TLR signaling pathway. In addition, *A. muciniphila* further elevates islet β-cell secretion, attenuates apoptosis and improves differentiation and the TLR signaling pathway on the basis of diet. Thus, the mechanism of the effect of *A. muciniphila* on pancreatic islets in pre-DM needs to be further studied to find a new target for the treatment of pre-DM.

## Data Availability

All relevant data and material are presented in this paper. The datasets generated during and/or analyzed during the current study are available from the corresponding author on reasonable request.

## Abbreviations

TLRs: Toll-like receptors
HFD: High-fat diet
pre-DM: Prediabetes mellitus
T2DM: Type 2 diabetes mellitus
IR: Insulin resistance
SPF: Specific pathogen-free
IPGTT: Intraperitoneal glucose tolerance test
FPG: Fasting plasma glucose
2h-PG: 2-hour postload plasma glucose
FINS: Fasting serum insulin
AUCG: The area under the curve of glucose
AUCINS: The area under the curve of insulin
ΔI30/ΔG30: Insulin/glucose 30 min ratio
HOMA-β: β cell function of homeostasis model assessment
HOMA-IR: Homeostasis model assessment of insulin resistance
LPS: Lipopolysaccharide
LBP: Lipopolysaccharide binding protein
GSIS: Glucose-stimulated insulin secretion
qRT‒PCR: Real-time fluorescence quantitative polymerase chain reaction
ELISA: Enzyme-linked immunosorbent assay
TUNEL: Terminal-deoxynucleotidyl transferase-mediated dUTP nick-end labeling
ASV: Amplicon sequence variant
SDs: Standard deviations
